# Knowledge and experiences of families regarding amber necklaces

**DOI:** 10.1186/s12906-023-04130-5

**Published:** 2023-09-02

**Authors:** Bekir Aktura, Mert Haci Dertli, Ege Hazal Ozen, Ezgi Onaran, Elnur Hashimov, Mine Basibuyuk, Nalan Karabayir

**Affiliations:** 1 Faculty of Medicine, Family Medicine Department, Istanbul Medipol University, Istanbul, Turkey; 2 International School of Medicine, Istanbul Medipol University, Istanbul, Turkey; 3 Faculty of Medicine, Istanbul Medipol University, Istanbul, Turkey; 4 Faculty of Medicine, Pediatrics Department, Istanbul Medipol University, Istanbul, Turkey; 5 Pediatrics Department, Istanbul Medipol University International School of Medicine, Istanbul, Turkey

**Keywords:** Amber necklaces, Suffocation, Teething, Risks

## Abstract

**Background:**

Amber necklaces have been used frequently to reduce the complaints of babies during teething. In this study, the knowledge and experience of families regarding the use of amber necklaces investigated.

**Methods:**

The structured questionnaire was applied face-to-face to parents with a 4–24 month old baby who applied to the paediatric outpatient clinic.

**Results:**

One hundred one families participating in the study reported that they used the amber necklace most frequently for restlessness (n = 72, 71.3%). Eighty- three% of families reported that the amber necklace was beneficial. It determined that 2% of the babies had suffocation and 2% had problems dispersing the grains.

**Conclusion:**

Although the parents think that the use of amber necklaces is effective during the teething period, they are not aware of the risks. It is important for healthcare professionals to inform their families about teething and especially the risks of using amber necklaces.

**Supplementary Information:**

The online version contains supplementary material available at 10.1186/s12906-023-04130-5.

## Background

During primary teeth eruption, parents often report local and systemic manifestations in their children. These may include gingival itching and inflammation, irritability, increased thumb sucking or putting objects in the mouth, loss of appetite, low-grade fever, hypersalivation, diarrhea, and insomnia [1, 2]. According to a meta-analysis of 16 studies, 70.5% of children between 0 and 36 months of age show signs and symptoms of teething during the eruption of the first teeth. The most frequent symptoms reported by parents include gingival irritation (86.81%), irritability (68.19%), and drooling (55.72%). The irritability of children experiencing discomfort and pain during teething periods is often a source of concern for parents [3].

Pharmacological and non-pharmacological methods are available to alleviate the distress children experience during tooth eruption. While medications such as analgesics can effectively reduce the pain associated with teething, some parents may be hesitant to use therapeutic approaches that could potentially threaten their child’s health [4]. Parents often use non-pharmacological methods to soothe their distressed children with teething symptoms, including remedies with mechanical actions on the gums such as biting on objects or foods and massaging the gums. A clinical study involving 270 children aged 8–36 months evaluated the perceived efficacy of five different non-pharmacological interventions for teething management by parents. According to the study, the most effective methods for reducing irritability were food for chewing (45.7%) and teething rings (42.1%), followed by cuddle therapy (17.1%), rubbing gums (15.8%), and cooling gums with a piece of ice (10.9%) [5].

Scientific evidence based on appropriate methodologies is still needed to demonstrate the efficacy of remedies with mechanical actions on the gums for their acute soothing and calming effect [6].

Several non-pharmacological methods based on popular and traditional beliefs have been used to relieve teething symptoms. Some dentists may recommend using teething gels containing benzocaine or choline salicylate to reduce pain. However, these chemical products should be used with caution due to the risk of methemoglobinemia, interference with the gag reflex, and intoxication. In other words, pharmacological products such as topical analgesics or systemic medications may lead to complications or have side effects [7]. To avoid these potential risks, some parents prefer to use safer non-pharmacological methods as remedies for teething problems. These may include homeopathic and natural remedies, behavioral therapy, chewing on clean, cool objects such as a chilled teething ring or rattle, chilled hard vegetables, or gingival massage with a cold, wet washcloth. The most commonly used methods to comfort babies are teether, teething gels and granules, herbal products such as clove oil, and oral antipyretics [3].

Amber, a natural resin, has been used for a wide variety of treatments in Ancient Greece, from the time of Hippocrates to the Middle Ages and even up to the present day, due to the belief that the succinic acid it contains has a healing effect [3]. Theories on the mechanism of action are based on the “bio-transmitter” and “electromagnetic” effects [8]. Another theory suggests that the succinic acid contained in amber beads is absorbed through the skin and exerts analgesia and anti-inflammatory effects [9]. Since various inflammatory cytokines such as Interleukin-1, Interleukin-10, and tumor necrosis factor increase during the teething period and cause symptoms, it is suggested that the anti-inflammatory effect of the amber necklace may relieve babies during the teething period [9].

In this study were investigated the knowledge and experience of parents regarding the use of amber necklaces during the teething period.

## Methods

The study was conducted between February 20, 2022, and March 5, 2022, at a pediatric outpatient clinic. With the Epi Info™ program, at least 96 sample sizes were determined in an unknown population with 10% margin of error and 95% confidence interval. Semi-structured questionnaires were conducted face-to-face by four researchers with 101 parents who had babies aged 4–24 months, had been using an amber necklace for at least one month, and agreed to participate in the study.

The parents were asked about their child’s age, the reason for using the amber necklace, when they started using it, who recommended it, whether it was useful, where it was purchased from, the duration of use, whether they were aware of the risks involved, any problems experienced during use, how it was cleaned, other methods used, and which method was considered to be the most effective. After completing the questionnaire, parents who were not aware of the risks were informed. The questionnaires were filled in by researchers (MHD, EHO, OE, HE) based on the parents answers.

Parents gave their oral and signed informed consent for participation, and the study received blinded statement approval from the institutional review board (E-10840098-772.02-1173).

Descriptive statistics were used to analyze the data, including mean, standard deviation, median, minimum, maximum, frequency, and ratio values. For the analysis of qualitative independent data, the Chi-square test and Fisher-Exact test were used. p-value of less than 0.05 was considered statistically significant. The analysis was conducted using the SPSS 22.0 software package.

## Results

A total of 101 families participated in the study. Of the babies aged 4–24 months, 44 (43.6%) were girls and 57 (56.4%) were boys, with a mean age of 17.90 ± 6.41 months. The average age at which babies started using amber necklaces was 5.40 ± 2.63 months and the average necklace use of babies was 12.5 ± 6.36 months. Socio-demographic data were shown in Table 1.

**Table 1:** General features

	Mean ± std min-max	Median
Age (month)	17.90 ± 6.41 5.0–24.0	19.0
Usage duration (month)	12.50 ± 6.36 1.0–23.0	14.0
Starting time (month)	5.40 ± 2.63 1.0–13.0	6.0
Gender/Age Group	N	%
Boy	57	56.4%
Girl	44	43.6%
≤ 12 months	25	24.8%
> 12 months	76	75.2%

The most common reasons for amber necklace use were restlessness (n = 72, 71.3%), gum itching (n = 56, 55.4%) and increased saliva secretion (n = 58, 57.4%). Amber necklaces were most commonly recommended to families by friends/relatives (n = 65, 64.4%) and purchased via the internet (n = 51, 50.5%). While 83.2% (n = 84) of the families stated that the amber necklace was beneficial, it was found that the amber necklace was very effective in 34.7% of the cases (Table 2).

No significant relation was found between the reason for the use of amber necklaces, gender, age groups and the positive effect of amber (p > 0.05) (Table 3).

**Table 2:** Data About Amber Necklace Use

		n	%
Information source	Physician	1	1.0
	Friend/relative	65	64.4
	Social media	35	34.6
Purchased from	Internet	51	50.5
	Store	31	30.7
	Gift	19	18.8
Reason of use*	Restlessness	72	71.3
	Increased salivation	58	57.4
	Itching of gums	56	55.4
	Crying	22	21.8
	Redness/swelling	16	15.8
	Anorexia	13	12.9
Usefulness	None	17	16.7
	A little	14	13.9
	Moderate	35	34.7
	Great	35	34.7
Cleaning method	Washing with soap and water	57	56.4
	While washing the baby	44	43.6
Being informed about risks	No	73	72.3
	Yes	28	27.7
Known risk by parent*	No risk	43	42.6
	Tangling on the neck	47	46.5
	Aspiration of the beads	24	23.8
	Skin Infection	5	5.0
Problems during usage	No	97	96.0
	Strangulation	2	2.0
	Dispersal of the beads	2	2.0

* Since; participants can indicate more than one reason for use. the total may be more than 100%

**Table 3:** The relationship between amber necklace effectivity. gender. age groups and reason for use

		Effectivity				
		Yes		No		
		n	%	n	%	p
Gender	Girl	7	15.9	37	84.1	0.828
	Boy	10	17.5	47	82.5	
Age group*	≤ 12 months	3	12.0	22	88	0.552
	> 12 months	14	18.4	62	81.6	
Purpose of use*	Crying	4	18.2	18	81.8	0.848
	Restlesness	14	19.4	58	80.6	0.269
	Anorexia**	4	30.8	9	69.2	0.225
	Itching of gums	9	16.1	47	83.9	0.820
	Redness/swelling**	1	6.3	15	93.8	0.296
	Increased salivation	7	12.1	51	87.9	0.137

*Chi- Square test. **Fisher-Exact test

Although, it was found that 2% of the babies had suffocation risk and 2% had the problem of dispersal of the beads, the rate of warning by the doctor/nurse about the risks of using amber necklaces was 27.7% (n = 28). Also, it was detected that 42.6% of parents did not know any risk of using amber necklaces (Table 2).

As, 56.4% (n = 57) of the families reported that they cleaned the necklaces by washing them with soap and water and 43.6% (n = 44) that they did the cleaning while washing the baby.

**Fig. 1:**
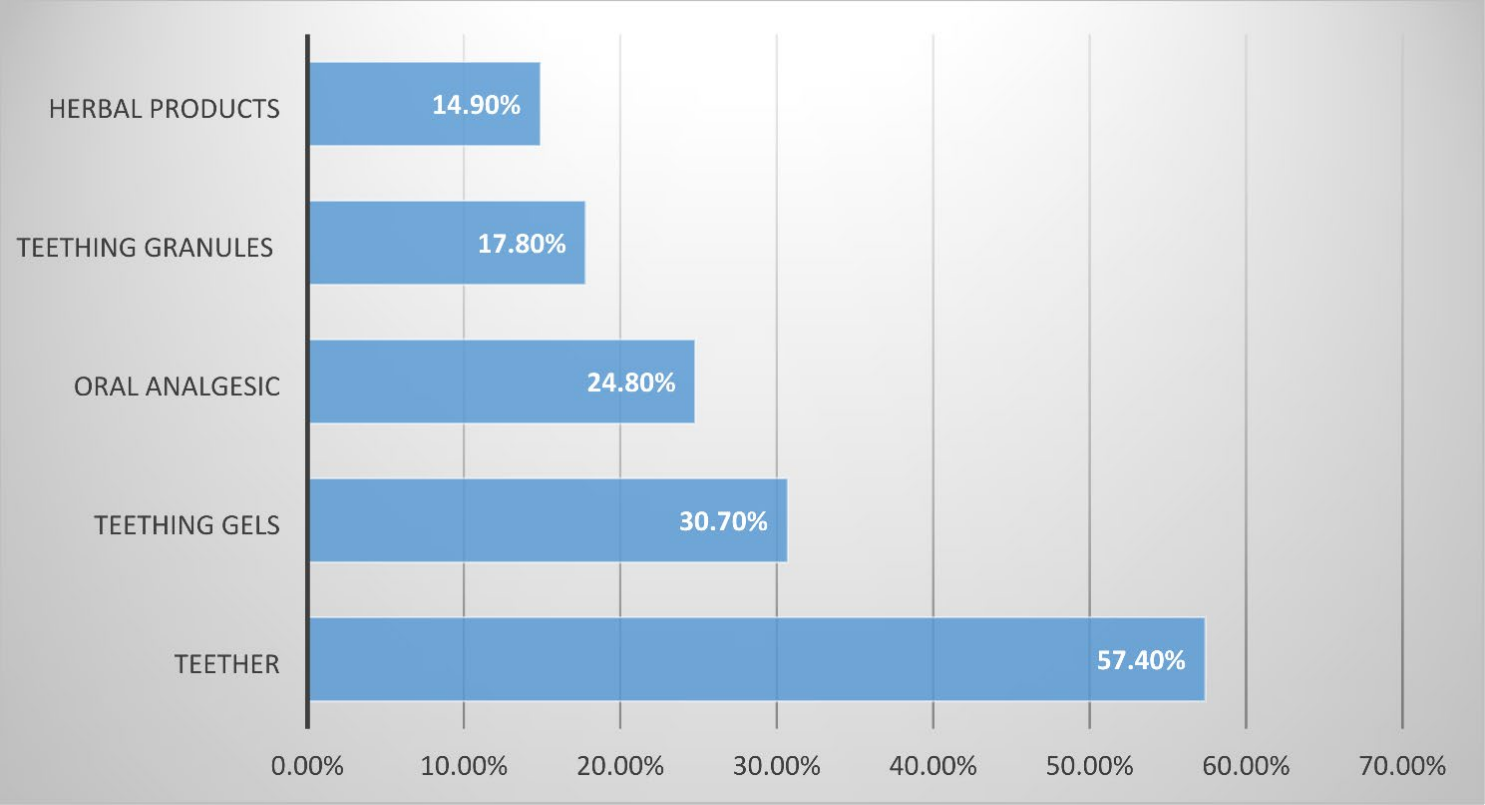
Freguency of methods other than amber necklaces

The rate of those who used other methods in addition to the amber necklace was found to be 85.1%. The methods used by families other than amber necklaces are respectively; teether (n = 58, 57.4%), teething gels (n = 31, 30.7%), oral analgesics (n = 25, 24.8%), teething granules (n = 18, 17%) ,8) and herbal products (n = 15, 14.9%) (Fig. 1). The average number of the methods used, including amber, was 2.46 ± 0.99. Parents stated that the most effective method was amber necklace (n = 28, 27.7%) and teething granules (n = 17, 16.8%) respectively (Table 4).

**Table 4:** The Method Considered by Parents to be The Most Effective

	n	%
Amber Necklace	28	27.7
Homepatic Remedies	17	16.8
Analgesics	15	14.9
Teether	15	14.9
Gels	13	12.9
Herbal Medicine	8	7.9
None of Above	5	5

## Discussion

Teething is a natural process, but parents often turn to drugs and treatments, such as gels, teething granules, oral antipyretics, and herbal products, to relieve their babies during this period. Amber necklaces have also become popular for this purpose in recent years.

Amber is a natural resin that contains succinic acid, which is thought to have healing properties [10]. Teething necklaces and bracelets were designed based on the hypothesis that amber would be effective in relieving symptoms related to inflammation during teething. In a study, it was reported that amber necklaces were most commonly used to prevent pain [11]. However, a study by Nissen et al. found no evidence of intact succinic acid being released and absorbed by human skin. Even if it were absorbed, it is unlikely to produce anti-inflammatory effects on inflammation mediators, as it would require a much higher temperature than that generated by simple contact between the stone beads and the child’s body [8].

In our study, the most common reason for using amber necklaces was restlessness (71.3%), followed by increased salivation (57.4%) and gingival itching (55.4%). The average age of starting to use an amber necklace was 5.40 ± 2.63 months in our study, compared to 4.2 months in the study conducted by Taillefer et al. [12]. Amber necklaces are often purchased online or given as gifts [11, 12]. However, in a study conducted in our country was found that physicians do not recommend the use of amber necklaces [13].

While some studies have shown that amber necklaces can benefit teething symptoms, others have found no scientific evidence to support their effectiveness. A study from Australia found that succinic acid was not released from the beads of the amber necklace [10]. Machet et al. determined that 33.3% of the cases benefited from amber necklaces at a high level and 40.7% at a moderate level [11]. In our study, 83.2% (n = 84) of the families reported that the amber necklace was beneficial, with 34.7% reporting it as highly effective and 16.8% reporting it as ineffective. There was no relationship between the effectiveness of the amber necklace and the gender or reason for using it.

However, the use of amber necklaces also poses significant safety risks, with choking being the leading cause of death for children under one year of age and among the top five causes of death for children aged 1 to 4 years. The primary risk associated with the use of amber necklaces is the possibility of suffocation. Choking occurs when necklaces are worn around a child’s neck, especially when they are unattended (during sleeping, etc) or if the child breaks the necklace and swallows the beads [14, 15]. Cases resulting in injury, suffocation, and death related to the use of amber necklaces have been reported in the literature [16–18]. In our study, aspiration risk was found as a result of 2% suffocation and 2% dispersion of beads.

In a study conducted in France, it was found that although life-threatening risks were mentioned in interviews with families of babies wearing amber necklaces, only 2 out of 13 families stopped using the necklace. In addition, 92% of the families were not informed about the risks by the sales representative [12]. In our study, we found that 72.3% of the families were not warned about the risks of using amber necklaces, and 42.6% were not informed about the risks. The fact that the parents participating in our study bought the amber necklace from places unrelated to health care (internet, store) may also be a reason why they were not warned about the risks of the amber necklace. According to the American Academy of Pediatrics (AAP), amber necklaces should be worn on the hand or ankle instead of the neck, and parents should not forget to remove the bracelet or necklace when the child is unattended, even for a short period of time. The necklace or bracelet should also be removed during sleeping (day or night) [15].

In a study about the infection risk of amber necklaces, bacterial colonization was found in the necklaces at a rate of 88.9%, despite regular cleaning [11]. This study reported that the median age for amber necklace usage was 10.7 months, and the median usage time was 4.1 months. However, the majority of infants in our study were older than 12 months and the median usage time was 14 months, and no infection was reported in our group. The fact that parents do not report an infection does not necessarily mean that the infection has not occurred.

In a study among Australian parents, it was found that teethers (65%) and paracetamol (60%) were the most commonly used methods during teething [19]. Acetaminophen or ibuprofen (80%) and a teether (59%) were the most commonly recommended methods by family physicians and pediatricians [19]. The most frequently used non-amber necklace methods during teething were a teether (57.4%), gel (30.7%), and analgesics (24.8%). In 27.7% of cases, the amber necklace was reported to be the most effective method.

## Conclusion

Although teething is a natural process, parents often seek different methods to comfort their babies during this period. Amber necklaces have become increasingly popular among parents, who report high satisfaction with their use compared to other methods. However, many parents are unaware of the potential risks associated with amber necklaces, such as choking or strangulation. It is important for health professionals to educate parents about these risks and advise them to remove the necklace while the child is asleep. However, we believe that amber necklace manufacturers should also indicate potential health hazards on the product’s packaging.

### Electronic supplementary material

Below is the link to the electronic supplementary material.

Supplementary Material 1

## Data Availability

The datasets used and/or analysed during the current study are available from the corresponding author on reasonable request.
