# Supplementary material for: Knowledge and experiences of families regarding amber necklaces
Source: BMC Complement Med Ther. 2023 Sep 2;23:306. doi: 10.1186/s12906-023-04130-5 (PMC10474688; doi:10.1186/s12906-023-04130-5)
Supplement: Supplementary file 1 — Supplementary Material 1 [file 12906_2023_4130_MOESM1_ESM.pdf]

1. Child's age (in months)
   ……………………………..
2. Gender
   a. Girl
   b. Boy
3. Reason for using amber necklace(Multiple answers can be selected)
   a. Restlessness
   b. Increased salivation
   c. Itching of gums
   d. Crying
   e. Redness/swelling
   f. Anorexia
4. Time to use (month)
   ………………………………………
5. İnformation source
   a. Physician
   b. Friend/relative
   c. Social media
6. Did it usefull?
   a. None
   b. A little
   c. Moderate
   d. Great
7. Where did you buy it?
   a. Internet
   b. Store
   c. Gift
   d. Pharmacy
8. How long was it used (months)
   ………………………………………
9. Is there any risk you know? (multiple selection possible)
   a. No risk
   b. Tangling on the neck
   c. Aspiration of the beads
   d. Skin Infection
10. Have you been warned about the risks by the physician/nurse?
    a. Yes
    b. No
11. Did you have any problems with usage?
    a. No
    b. Strangulation
    c. Dispersal of the beads
    d. Skin Infection
12. How do you do your cleaning?
    a. Washing with soap and water
    b. While washing the baby
13. Methods used during teething (may be more than one)
    a. Amber Necklace

b. Homepatic Remedies
c. Analgesics
d. Teether
e. Gels
f. Herbal Medicine
g. None

14. What do you think is the most effective method?
    a. Amber Necklace
    b. Homepatic Remedies
    c. Analgesics
    d. Teether
    e. Gels
    f. Herbal Medicine
    g. None
